# Hypoxia-Inducible Factor Prolyl Hydroxylase Inhibitor Prevents Steroid-Associated Osteonecrosis of the Femoral Head in Rabbits by Promoting Angiogenesis and Inhibiting Apoptosis

**DOI:** 10.1371/journal.pone.0107774

**Published:** 2014-09-22

**Authors:** Lihong Fan, Jia Li, Zefeng Yu, Xiaoqian Dang, Kunzheng Wang

**Affiliations:** Department of Orthopedics, The Second Affiliated Hospital of Xi'an Jiaotong University, Xi'an, Shaanxi, China; Van Andel Institute, United States of America

## Abstract

The purpose of this study was to investigate the preventive effect of ethyl 3,4-dihydroxybenzoate(EDHB) on steroid-associated femoral head osteonecrosis(ONFH) in a rabbit model. New Zealand white rabbits were randomly divided into two groups (prevention group and model group), each containing 24 rabbits. Osteonecrosis was induced by lipopolysaccharide(LPS) combined with methylprednisolone(MPS). The prevention group received an intraperitoneal injection of EDHB at 50 mg/kg body weight every other day starting three days before establishing rabbit models of osteonecrosis, for a total of nine doses. Osteonecrosis was verified by haematoxylin-eosin (HE) staining. The expression of HIF-1α and VEGF was analyzed by immunohistochemistry. Angiogenesis, apoptosis and microstructural parameters were also analyzed. The rabbit models of osteonecrosis were successfully established and observed by HE staining. Histopathological observations indicated that EDHB reduced the rate of empty lacunae and the incidence of osteonecrosis. Immunohistochemical staining for HIF-1α and VEGF suggested that EDHB therapy inhibited degradation of HIF-1α and promoted expression of VEGF. Ink artery infusion angiography and microvessel density analysis revealed that there were more microvessels in the prevention group than in the model group. The TUNEL apoptosis assay suggested that EDHB intervention could reduce the number of apoptotic cells in avascular osteonecrosis of the femoral head. Micro-CT scanning indicated that the treatment group had better microstructural parameters than the model group. EDHB prevents steroid-associated osteonecrosis of the femoral head in rabbits by promoting angiogenesis and inhibiting apoptosis of bone cells and hematopoietic tissue.

## Introduction

Steroid-associated osteonecrosis of the femoral head(ONFH), which is characterized by trabecular bone necrosis and bone marrow necrosis, is an aseptic and ischemic disease caused by long-term glucocorticoid use or heavy use over a short period of time. The pathogenesis of steroid-associated ONFH is not clearly understood. It is generally agreed that the final common pathway is interruption to the bone microcirculation and failure to deliver necessary nutrients, leading to the death of osteocytes and fat cells with resultant bone marrow edema and ultimately, destruction of the bone architecture [1]. Steroid-associated ONFH is a common disease which is both progressive and refractory. It accounts for the majority of the ONFH [2] and brings great suffering to families and society. If not treated properly and promptly, 80% of the femoral heads will collapse once the osteonecrosis process begins [3]. When the disease reaches this point, the only choice is arthroplasty. Because most of the affected patients are young individuals, a considerable number of them will need revision surgeries. Therefore, it is highly desirable to develop promising methods to prevent steroid-associated ONFH, slow down the collapse of the articular surface and avoid or delay the need for hip arthroplasty.

Numerous measures have been taken to prevent steroid-associated ONFH and slow down its progress including conservative treatments such as decreasing weight bearing, lipid-lowering drugs, anticoagulants, anti-osteoporosis drugs, electromagnetic or electrical stimulation, extracorporeal shockwave therapy, hyperbaric oxygen treatment, as well as surgical management such as core decompression and autologous bone marrow mononuclear cells transplantation. Unfortunately, none of these methods is 100% successful, and the results have been variable [4], [5].

Hypoxia-inducible factors (HIFs) are DNA-binding transcription factors that activate a series of hypoxia associated genes under hypoxia to trigger adaptive responses to decreased oxygen tension. Approximately 100 target genes of HIFs have been identified, including vascular endothelial growth factor(VEGF), hemeoxygenase-1, and the glucose transporter protein 1 [6]. HIF target genes are particularly relevant to angiogenesis, erythrocytopoiesis and cytoprotection [6]. Previous studies have shown that activating the HIF pathway via pharmacological or genetic approaches promotes angiogenesis, erythropoiesis, cell proliferation, and cell survival [7]–[10]. Other recent studies indicated that HIF-1α and its target gene VEGF play an important role in angiogenic-osteogenic coupling during bone regeneration [11]. These findings implicate that activating the HIF pathway may be a novel and simple way to prevent the development of steroid-associated ONFH by promoting angiogenesis, bone regeneration and cytoprotection. Ethyl 3,4-dihydroxybenzoate(EDHB) is a small molecular drug and is known as a inhibitor of hypoxia-inducible factor prolyl hydroxylase domain proteins(PHDs). Under normoxia, PHDs hydroxylate particular proline residues within the oxygen dependent degradation domain of HIF-1α. The hydroxylated HIF-α then binds to the Von Hippel-Lindau tumor suppressor, leading to the degradation of HIF-α [12]. EDHB has been found to stabilize HIF-1α expression under normoxic conditions in vitro and in vivo [13]–[15].

As discussed earlier, the final common pathway of steroid-associated ONFH is an interruption to the bone microcirculation. Considering that EDHB stabilizes HIF-1α expression and can acquire beneficial aspects of the HIF system, systemically administering EDHB to induce angiogenesis, bone regeneration and cytoprotection might be a promising way to prevent steroid-associated ONFH. Furthermore, to the present authors' knowledge, there is no report on preventing steroid-associated ONFH by systemically administrating hypoxia-inducible factor prolyl hydroxylase inhibitor. Therefore, the aim of the present study was to investigate the preventive effect of EDHB on steroid-associated ONFH and explore the effects of EDHB on angiogenesis and apoptosis in a rabbit model.

## Materials and Methods

### Establishment of animal model and treatment protocol

We used male New Zealand white rabbits used were from the Experimental Animal Center of Xi'an Jiaotong University, China. All experimental animals were housed under standard conditions, and the experimental protocols were approved by the Animal Ethical Committee of the Xi'an Jiaotong University and conducted in accordance with the NIH Guide for the Care and Use of Laboratory Animals. All efforts were made to minimize the animals' suffering. Fourty-eight male New Zealand white rabbits (age: 28 weeks; body weight: 2.5–3 kg) were randomly divided into two groups: the prevention group (LPS, MPS plus EDHB) consisting of 24 rabbits, and the model group(LPS plus MPS), also consisting of 24 rabbits. Osteonecrosis was induced using methods presented in the previously published protocols described by Ling Qin et al. [16]. ON gradually developed 6 weeks after injection of MPS. Briefly, one injection of 10 µg/kg body weight of LPS(Sigma, St. Louis, MO, USA) was given intravenously. 24 hours later, three injections of 20 mg/kg body weight of MPS(Pfizer, USA) were given intramuscularly, at a time interval of 24 h. Concurrently, the prevention group received intraperitoneal injection of 50 mg/kg body weight of EDHB(Sigma, St. Louis, MO, USA) every other day begining three days before establishing the rabbit models of osteonecrosis for a total of nine doses; The model group received the same amount of vehicle. At the time of the hormone injection, each animal was injected intramuscularly with 200 000 U of penicillin. Six weeks later, 5 rabbits were used for intra-arterial ink perfusion and the others were used for micro-CT scanning and histopathological analysis.

### Micro-CT scanning

Bone structure was evaluated non-invasively by micro-CT(eXplore Locus SP, GE, USA) using a high-resolution system(MS-8) 6 weeks after the induction of osteonecrosis. Data sets with isotropic 14 µm voxel spacing were acquired at 0.5°steps over a total rotation of 360°at 80 kVp with an exposure time of 3000 ms/frame. A polycarbonate calibration phantom containing 2.3 mm diameter cylinders of air, water and SB3, which is a hydroxyapatite (HA) mimicking material was scanned to scale the values of CT attenuation to bone mineral density (BMD). Images were reconstructed into 3-D volumes using a Reconstruction Utility with 16-bit gray levels. In the trabecular region of the femoral head bone a 15 mm^3^ region of interest (ROI) was selected using a semiautomatic contouring method in the center of the femoral head. Microstructural parameters of ROI including bone mineral density(BMD; expressed as mg/cm^3^), tissue mineral density(TMD; expressed as mg/cm^3^), bone volume fraction(BVF; bone volume/total volume of bone [BV/TV]; expressed as a percent), trabecular number (Tb.N; expressed as 1/mm), trabecular thickness (Tb.Th; expressed as mm), and trabecular separation(Tb.Sp; expressed as mm) were calculated from the 3-D images using the Advanced Bone Analysis software (GE Health Care). Thresholds for each specimen were chosen to separate bone from marrow by determining the HU value that maximized the between class variance of water and bone values in the micro-CT scans [17].

### Intra-artery ink perfusion

To measure the blood supply of the femoral head, selective vascular perfusion with Indian ink was performed 6 weeks after the induction of osteonecrosis. The animals were anesthetized by an intraperitoneal injection of pentobarbital sodium. The abdominal aorta and inferior vena cava were exposed and ligated immediately. A tube was inserted into the abdominal aorta distal to the site of ligation and used to infuse ink. Another tube was distally inserted in the inferior vena cava for drainage. The abdominal aorta was irrigated with heparinized saline (25,000 units in 250 ml of 0.9% sodium chloride), until the liquid flowed freely from the inferior vena cava and was clear. The abdominal aorta was then injected continuously with a solution of 10% gelatin/Indian ink (20 g of gelatin in 100 ml of Indian ink and 100 ml of water) at a pressure of 90 mmHg, until the skin of the bilateral crura and nails were uniformly black. The animals were then euthanized. 24 hours after the refrigeration at 4°C, the bilateral thighbones were dissected and harvested. The samples were fixed, decalcified, embedded, cut into 25-µm-thick slices, and stained by hematoxylin and eosin. The configuration of the femoral head, blood distribution, and growth condition were observed under a stereomicroscope. The perfusion ratio of was calculated using the Image-Pro Plus 6.0 image analysis software. Ratio of perfusion was defined as the ratio of the area of inked artery to the area of the entire femoral head [18].

### Histopathology

After the animals were euthanized 6 weeks after the induction of osteonecrosis, the bilateral femoral heads were removed, observed by macrography, fixed with 10% neutral buffered formalin, decalcified with 10% ethylenediaminetetraacetic acid (EDTA), then embedded in paraffin, and cut along the coronal plane into 4-µm-thick sections. Sections were stained with hematoxylin-eosin. Changes in the periosteum, cartilage, trabeculae, and hematopoietic organization were observed. The presence or absence of osteonecrosis was determined by two researchers who were blind to the identity of the prevention group. A positive diagnosis of osteonecrosis was determined based on the diffuse presence of empty lacunae or pyknotic nuclei of osteocytes within the bone trabeculae, accompanied by surrounding bone marrow cell necrosis or fat cell necrosis. The number of rabbits that developed osteonecrosis, the source of the bone tissue, and the incidence of osteonecrosis was calculated. During the observation of the trabeculae at 200 times magnification, 10 fields were randomly chosen and 50 bone lacunae were counted in each field. The number of empty bone lacunae was counted, and the proportion of empty bone lacunae was calculated. During the observation of the fat cells in bone marrow, the average bone marrow fat cell size was calculated using Image-Pro Plus 6.0 software. The average bone marrow fat cell size was defined as an average of the greatest diameters of 100 fat cells in 4 randomly-selected non-necrotic fields.

### Immunohistochemistry

Two days after the first intraperitoneal injection of EDHB, HIF-1α and VEGF expression was measured by immunohistochemistry using antibodies against either HIF-1α or VEGF(Upstate Biotechnology, USA). 6 weeks after the induction of osteonecrosis, immunohistochemistry for CD31 (Upstate Biotechnology, USA), Bcl-2(Santa Cruz Biotechnology, USA) and caspase-3(Santa Cruz Biotechnology, USA) was also performed to measure blood vessels and apoptosis-related factor expression in the necrotic area of each group.. Primary antibodies against HIF-1α, VEGF, CD31, Bcl-2 and caspase-3 were used. The VEGF primary antibody is a mouse anti-rabbit antibody that recognizes 121, 165, and 189 amino acid isoforms of rabbit VEGF. A goat anti-mouse IgG biotinylated antibody diluted in a buffer was used as a secondary antibody. Briefly, endogenous peroxidase activity was deactivated by immersing sections in 3% hydrogen peroxide in 0.01 M PBS for 10 min and rinsing them several times in PBS. After being blocked with 10% goat normal antiserum(Vector, USA) for 30 min at room temperature, sections were treated with a primary antibody overnight at 4°C and incubated with the biotinylated secondary antibody for 30 minutes, followed by streptavidin peroxidases for 30 min at room temperature. Immunoreactivity was revealed by incubating the sections in a chromogen solution containing diaminobenzidine (DAB) and 0.1% hydrogen peroxide in the dark. Finally, the sections were treated with hematoxylin and mounted. As a negative control for staining, PBS solution or normal nonimmune mouse IgG was used in place of the primary antibody, while the remaining steps were identical. The positive staining images were quantitatively analyzed using Image-Pro Plus 6.0 software and calculating the mean optical density. Ten random fields in the bone marrow cavities were selected and the positive staining was quantitated based on integrated optical density (IOD). The corresponding cavity area was also measured. The mean optical density was defined as the ratio of integrated optical density to the corresponding cavity area.

### Quantification of microvessel density

Microvessel density (MVD), a measure of angiogenesis, was determined by light microscopy after immunostaining sections with anti-CD31 antibodies according to the procedure described by Weidner et al. [19]. Microvessels were counted on a 200× field. Any single endothelial cell or cluster of endothelial cells clearly separated from adjacent microvessels was considered one countable microvessel. Ten representative areas of the section were counted. The evaluation was performed by two researchers who were blind to the identity of the prevention group.

### TUNEL apoptosis detection

Terminal deoxynucleotidyl transferase-mediated dUTP nick end labeling (TUNEL) assays were used to observe the presence and location of apoptosis. The TUNEL detection kit (Promega Co., Ltd., Beijing, China) was used according to the manufacturer's instructions. The cells whose nuclei were brown or brown-yellow or whose cytoplasm included a few brown or brown-yellow granules were interpreted as positive. The sections were observed under 200× magnification and the apoptosis rate (number of TUNEL-positive cells/number of all cells) was calculated in each section by two researchers who were blind to ensure that all of the work was honestly reported. Besides, morphological changes characteristic of apoptosis were examined carefully to minimize ambiguity regarding the interpretation of positive results.

### Statistical analysis

All data were described as the mean ± standard deviation. Independent-samples t test was performed to analyze the statistical data, and the Chi-square test was performed for the count data. The SPSS 17.0 software was used for analysis. P<0.05 was considered to be statistically significant.

## Results

### EDHB reduced the rate of empty lacunae, the average bone marrow fat cell size and the incidence of osteonecrosis

**Figure 1:**
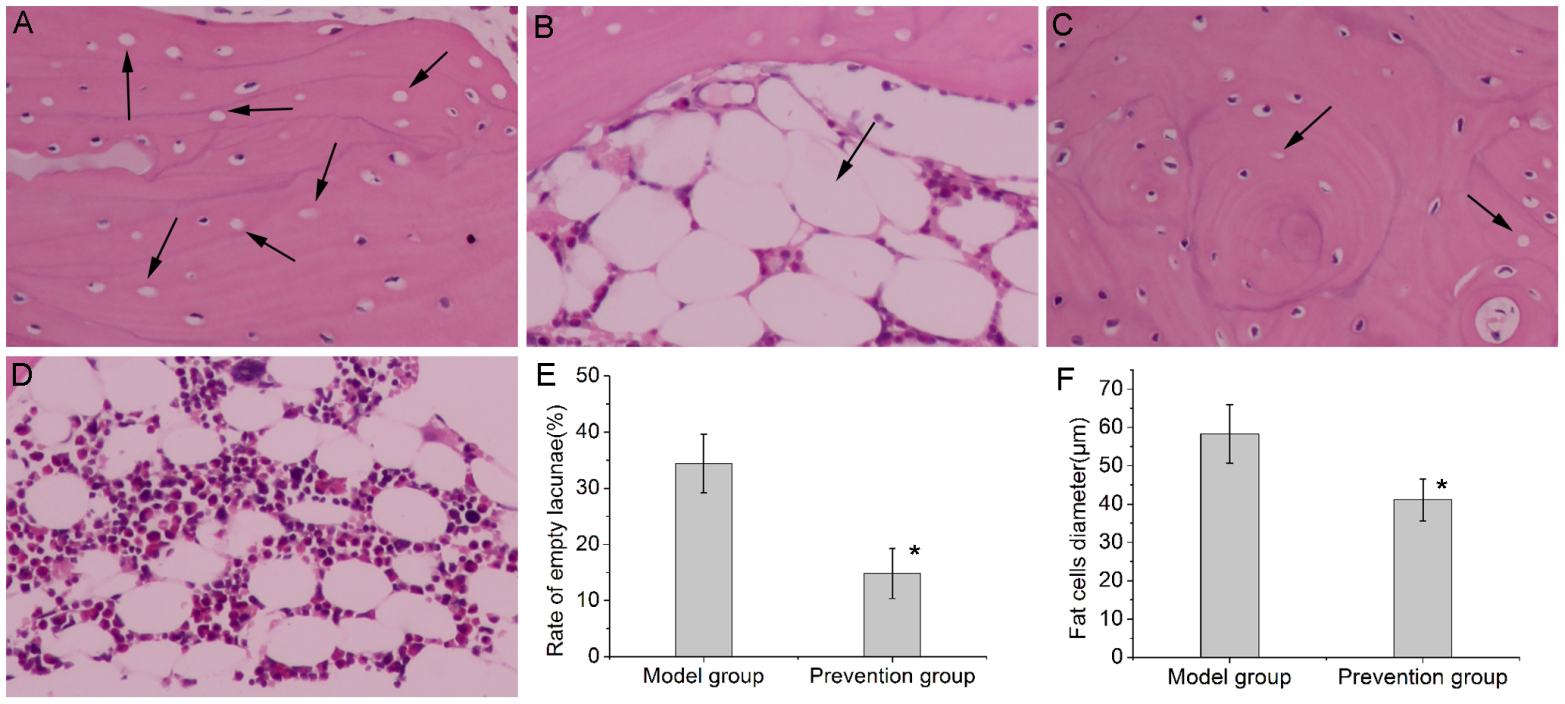
Histopathological observations (200×magnification). (A). Obvious osteonecrosis was observed in the model group. Osteocytes in the bone trabeculae showed pyknosis and empty lacunae. (B). The diameter of fat cells in the marrow of the model group significantly increased. (C). In the prevention group, fewer empty lacunae were observed. (D). Compared with the model group, the diameter of fat cells in the prevention group decreased. (E and F). Bar graph shows the rate of empty lacunae and diameter of fat cells. Both the rate of empty lacunae and diameter of fat cells in the prevention group was significantly lower than that in the model group and the significance was statistically significant(P<0.05). The asterisk (*) shows statistically significant difference between the two groups. Empty lacunae and enlarged fat cells are shown by arrows.

During the establishment of steroid-associated femoral head osteonecrosis model, four rabbits in the model group died of infection and were excluded from the evaluation. Osteonecrosis in the model group was obvious(Figure 1A and 1B). Bone cells in the bone trabeculae showed pyknosis and empty lacunae. The marrow tissue also had necrotic changes of hematopoietic and fat cells. The nuclei of bone marrow cells displayed pyknosis and stained acidophilic. The cellular structure of some fat cells collapsed. In the prevention group, slight osteonecrosis was observed with fewer empty lacunae and necrotic medullary hematopoietic cells and fat cells(Figure 1C and 1D). 17 of the 20 rabbits in the model group and 6 of the 24 rabbits in the experimental group developed osteonecrosis. EDHB reduced the incidence of osteonecrosis (P<0.05, Pearson chi-square test). The rate of empty lacunae in the prevention group was 14.8±4.4%(Figure 1C), which was significantly lower than the rate of 34.4±5.2% in the model group(P<0.05). The average bone marrow fat cell size in the model group was 58.3±7.6 µm. Compared with the model group, the diameter of fat cells in the prevention group(41.1±5.4 µm) decreased(P<0.05).

### EDHB therapy inhibited degradation of HIF-1α and promoted expression of VEGF

**Figure 2:**
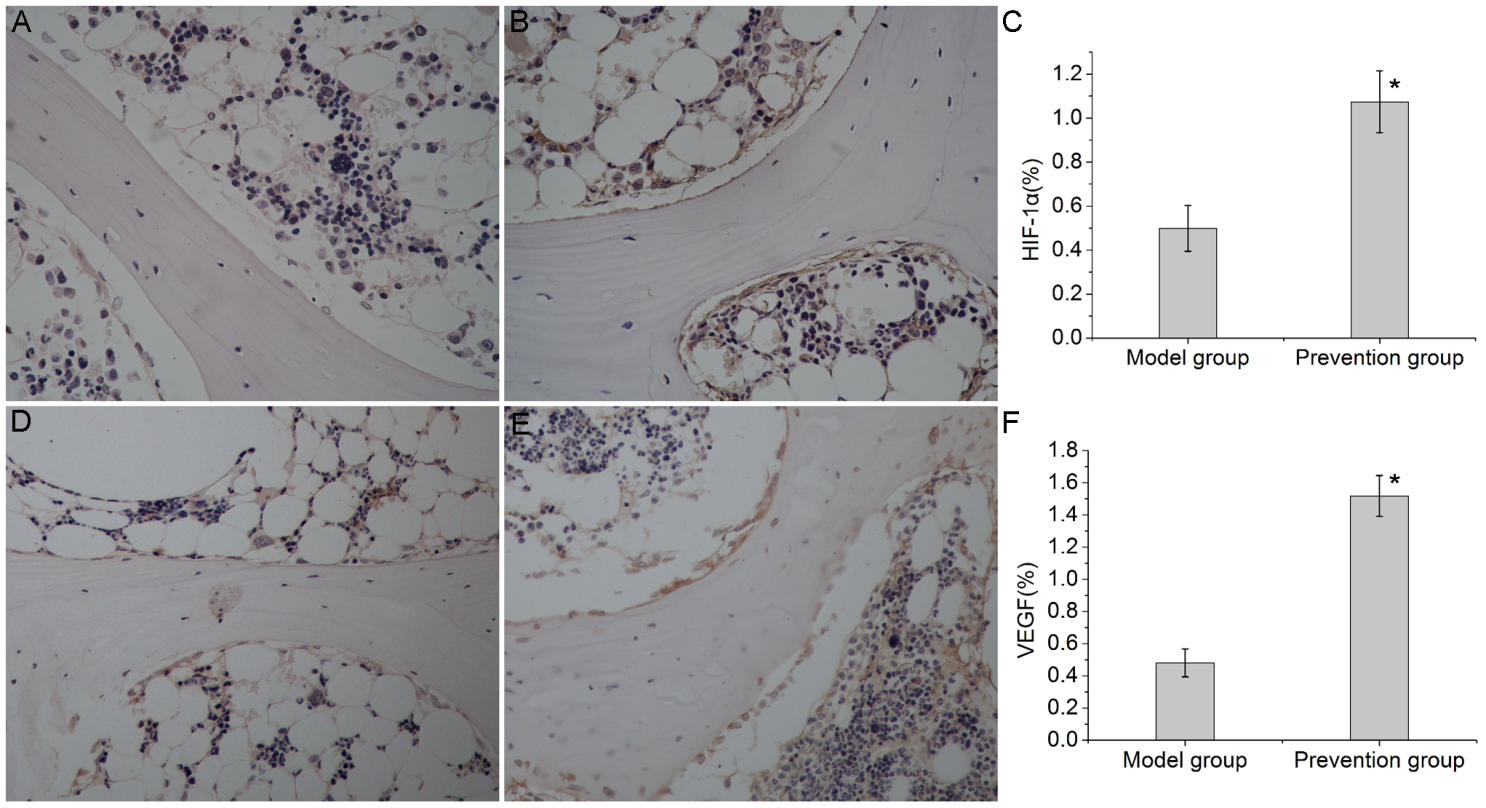
Immunohistochemical staining and semi-quantitative analysis of HIF-1α and VEGF expression(200×magnification). HIF-1α and VEGF immunoreactivity were mainly observed in the osteoblasts and endothelial cells in both groups. (A). In the model group, weak immunoreactivity of HIF-1αwas found in the osteoblasts and endothelial cells. (B). After treatment with EDHB, the osteoblasts and endothelial cells showed a significantly high level of HIF-1αimmunoreactivity in the prevention group. (C) and (F). Bar graphs represent the mean optical density of HIF-1α and VEGF in bone marrow. Semi-quantitative analysis was based on at least 10 fields per section. The asterisk (*) shows statistically significant difference between the groups. (D). In the model group, weak immunoreactivity of VEGF was found in the osteoblasts and endothelial cells. (E). After treatment with EDHB, the osteoblasts and endothelial cells showed a significantly high level of VEGF immunoreactivity in the prevention group.

HIF-1α and VEGF expression was measured by immunohistochemistry two days after the first intraperitoneal injection of EDHB. The HIF-1α protein was expressed in the cytoplasm and nuclei, while VEGF protein was expressed in the cytoplasm. HIF-1α and VEGF immunoreactivity were mainly observed in the osteoblasts and endothelial cells in both groups(Figure 2). In the femoral heads that were not treated with EDHB, a slight HIF-1αand VEGF immunoreactivity(HIF-1α-IR, VEGF-IR) was found in the osteoblasts and endothelial cells (Figure 2A and 2D). In the prevention group, the osteoblasts and endothelial cells showed an increased level of HIF-1α-IR and VEGF-IR (Figure 2B and 2E). These findings suggested that the HIF-1α pathway was activated after EDHB therapy.

### EDHB promoted angiogenesis

**Figure 3:**
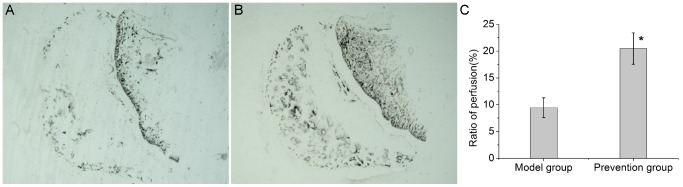
Ink artery infusion angiography of the femoral head and the ratio of perfusion. (A). Few blood vessels were found in the model group. (B). Compared with the model group, the prevention group had more ink-stained blood vessels. (C). Bar graph shows the ratio of perfusion in the femoral head. The ratio of perfusion in the prevention group was significantly higher than that in the model group. The asterisk (*) shows statistically significant differences between the two groups.

Microvessels in the femoral head were analyzed by ink artery infusion angiography and immunohistochemical staining for CD31. In the model group, few capillaries were observed to form a sparse network in the medullary cavity(Figure 3A). In the prevention group, there were more ink-stained capillaries forming a patent network(Figure 3B). The ratio of perfusion was 20.49±2.93% in the prevention group(Figure 3C), significantly higher than the rate of 9.45±1.84% observed in the model group(P<0.01, n = 10).

**Figure 4:**
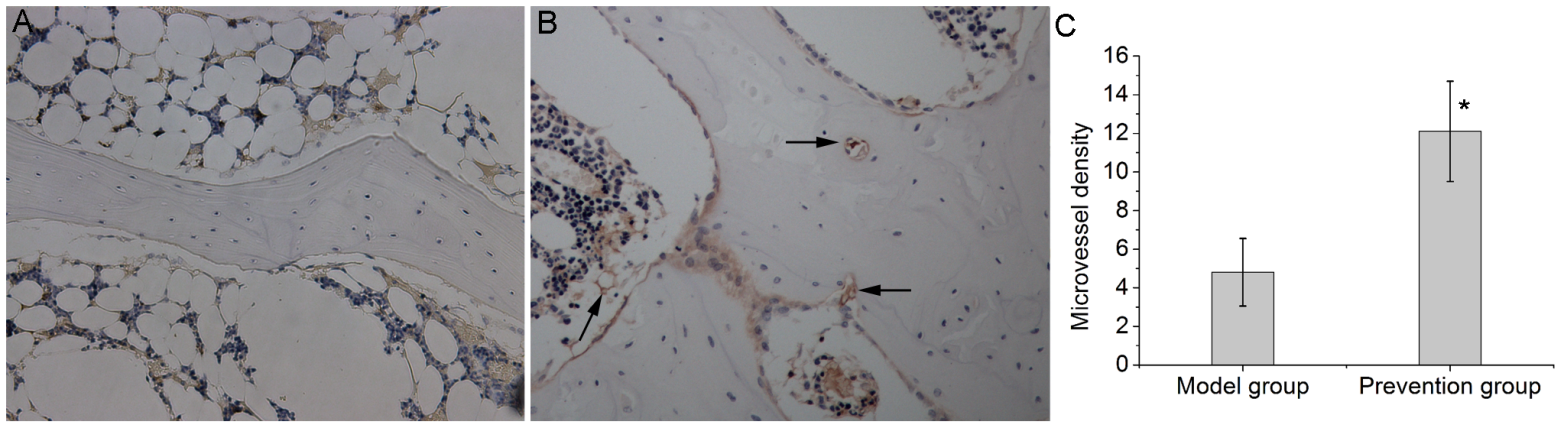
Immunohistochemical staining of CD31(200×magnification) and quantification of microvessel density. (A). There were only a few microvessels in the subchondral bone of the necrotic femoral heads in the model group. (B). The prevention group showed increased microvessel density after EDHB treatment. (C). Bar graph shows the comparison between the model group and the prevention group. The microvessel density in the prevention group was significantly higher than that in the model group(P<0.05). The asterisk (*) shows statistically significant differences between the two groups. The arrows pointed to a lot of microvessel in the prevention group.

After immunostaining for CD31, microvessel density (MVD) was calculated on a 200× field. Any single endothelial cell or cluster of endothelial cells clearly separated from adjacent microvessels was considered as one countable microvessel. Only a few microvessels in the subchondral bone of the necrotic femoral heads were observed in the model group(Figure 4A). In the prevention group, significantly higher numbers of microvessels in the subchondral bone were present(Figure 4B). There were significant statistical differences in MVD between the two groups(Figure 4C).

### EDHB inhibited apoptosis and expression of caspase-3 and induced expression of bcl-2

**Figure 5:**
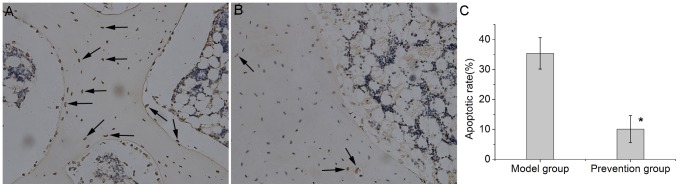
TUNEL apoptosis detection(200×magnification). (A). Extensive TUNEL-positive cells, including osteocytes, chondrocytes and bone-marrow cells were observed in the femoral heads of the model group. (B). In the prevention group, relatively lower numbers of apoptotic cells were observed. (C). Bar graph shows the comparison of apoptotic rate between the model group and the prevention group. In the prevention group, the apoptotic rate was significantly lower than that in the model group(P<0.05). The asterisk (*) shows statistically significant differences between the two groups. The arrows indicated TUNEL-positive cells.

In the model group, apoptosis was observed in various cell types including osteocytes, chondrocytes and bone marrow cells(Figure 5A). The mean apoptotic rate in the model group was 35.4±5.3%. A relatively lower number of TUNEL-positive osteocytes and bone-marrow cells(Figure 5B), and a lower mean apoptotic rate 10.1±4.5% was observed in prevention group. There were significant statistical differences(Figure 5C) between the two groups (P<0.05). These findings suggested that apoptosis occurred in the early stage of steroid-associated osteonecrosis and that EDHB intervention could prevents such changes.

**Figure 6:**
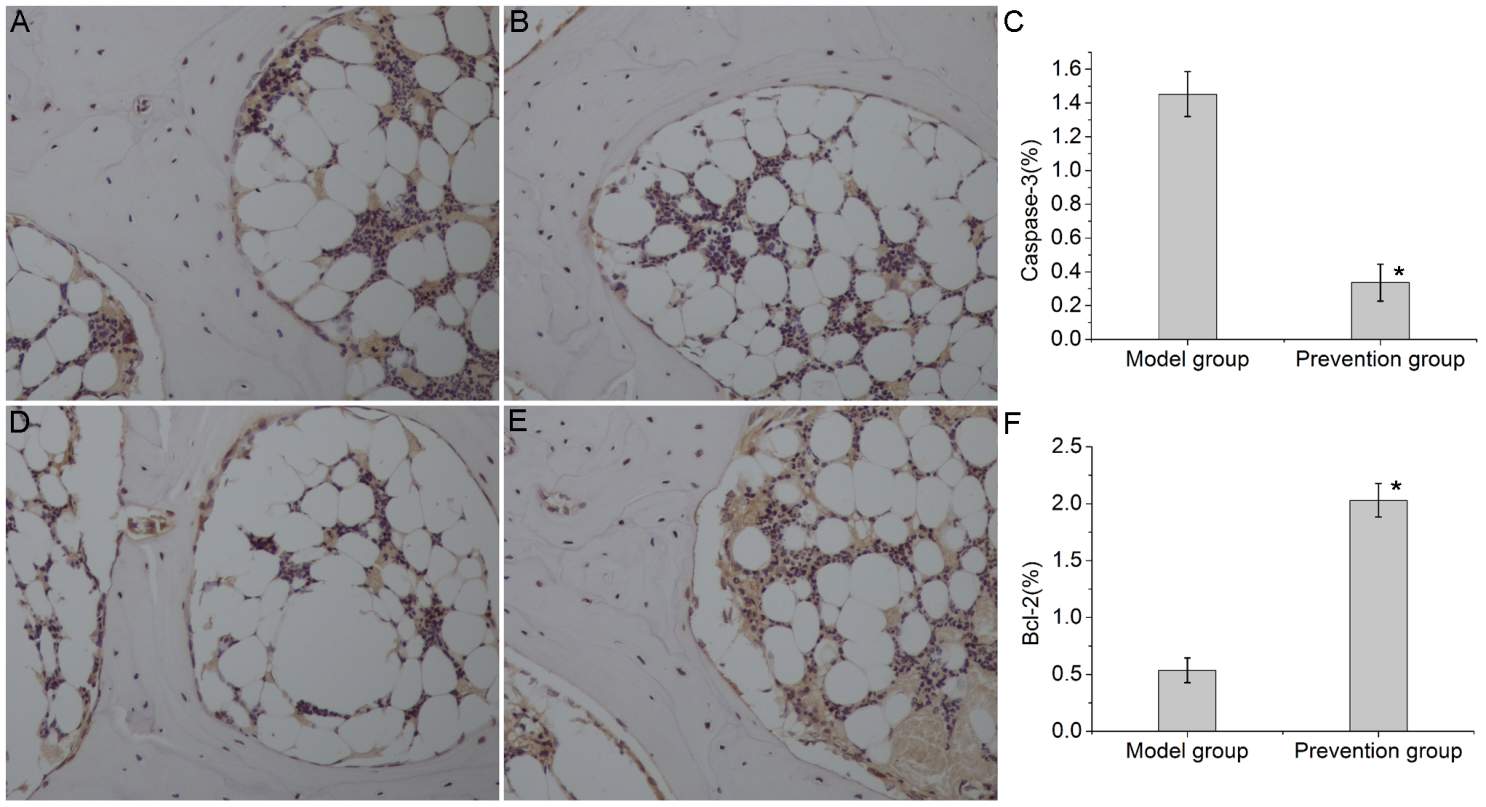
Immunohistochemical staining and semi-quantitative analysis of caspase-3 and Bcl-2(200×magnification). Caspase-3 and Bcl-2 immunoreactivity were observed in the cell cytoplasm of cartilage, trabecular bone and bone marrow. (A). In the model group, relatively high immunoreactivity of caspase-3 was observed. (B). In the prevention group, weak immunoreactivity of caspase-3 was found. (C) and (F). Bar graphs represent the mean optical density of caspase-3 and Bcl-2 in bone marrow. Semi-quantitative analysis was based on at least 10 fields per section. The asterisk (*) shows statistically significant differences between the two groups. (D). In the model group, low immunoreactivity of Bcl-2 was observed. (E). In the prevention group, higher immunoreactivity of Bcl-2 was found.

The expression of caspase-3and Bcl-2 was analyzed by immunohistochemical staining. Bcl-2 and caspase-3 were distributed in the cell cytoplasm of cartilage, trabecular bone and bone marrow. The immunoreactivity of caspase-3 was intense in the model group and weak in the prevention group (Figure 6A, 6B and6C). In contrast, the immunoreactivity of Bcl-2 was weak in the model group and intense in the prevention group (Figure 6D, 6E and 6F).

### EDHB ameliorated microstructure of the femoral head

**Figure 7:**
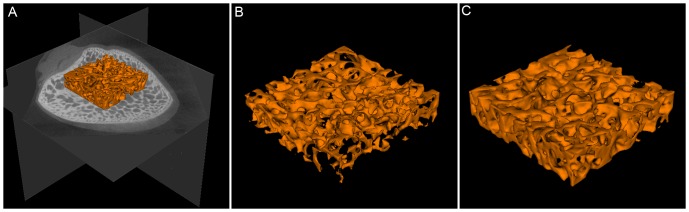
Representative three-dimensional micro-CT scanning images. (A). A region of interest(ROI) in the center of the femoral head was selected. (B). The magnified region of interest(ROI) in the femoral head of the model group. It showed that the trabecular bone in the model group was irregular and relatively thin. (C). The magnified region of interest(ROI) in the femoral head of the prevention group. The ROI in the prevention group showed better three-dimensional structure and structural integrity of the trabecular bone.

A region of interest(ROI) in the center of the femoral head was selected(Figure 7A). In the model group, the trabecular bone of the ROI in the femoral head was irregular and relatively thin(Figure 7B). The ROI in the prevention group showed a better three-dimensional structure and structural integrity of the trabecular bone(Figure 7C). The prevention group had better microstructural parameters than the model group (Table 1). The prevention group had higher values of BMD, TMD, BVF, Tb.N, and Tb.Th and a lower value of Tb.Sp than those in the model group(P<0.05 for all variables).

**Table 1:** The comparison of microstructural parameters of region of interest in the femoral head.

Microstructural parameters	BMD	TMD	BVF	Tb.N	Tb.Th	Tb.Sp
Model group(n = 10)	362.92±20.79	617.56±12.91	27.07±4.36	2.91±0.19	0.091±0.013	0.245±0.031
Prevention group(n = 10)	402.04±31.07	632.79±13.70	32.12±3.62	3.13±0.18	0.105±0.013	0.210±0.028
Statistical significance^a^	P<0.01	P<0.05	P<0.05	P<0.05	P<0.05	P<0.05

BMD, bone mineral density, expressed as mg/cm3; TMD, tissue mineral density, expressed as mg/cm3; BVF, bone volume fraction, bone volume/total volume of bone, expressed as a percent; Tb.N, trabecular number, expressed as 1/mm; Tb.Th, trabecular thickness, expressed as mm; Tb.Sp, trabecular separation, expressed as mm.

a Statistical significance was evaluated by independent-samples t test.

## Discussion

Steroid-induced osteonecrosis of the femoral head(ONFH) accounts for the majority of nontraumatic osteonecrosis of the femoral head. It occurs in patients who receive high-dose corticosteroid therapy for the treatment of diseases including systemic lupus erythematosus, inflammatory bowel disease, and also for immunosuppression after renal transplants. It tends to occur in relatively young patients. HIFs are crucial mediators of the adaptive cell response to hypoxia. The HIF pathway plays important roles in angiogenesis, erythrocytopoiesis, cytoprotection and angiogenic-osteogenic coupling. The aim of our study was to test whether EDHB, a small-molecule prolyl hydroxylase inhibitor (PHI) which inhibits the degradation of HIF-1α, prevents the steroid-associated osteonecrosis of the femoral head.

In our study, 17 of the 20 rabbits in the model group and 6 of the 24 rabbits in the experimental group developed osteonecrosis. Histopathological observations indicated that EDHB reduced the rate of empty lacunae, the diameter of fat cells and the incidence of osteonecrosis in accordance with the increased numbers of microvessels and the decreased numbers of apoptotic cells. In addition, micro-CT scanning indicated that EDHB ameliorated the microstructure of the femoral head.

EDHB, which is known as an inhibitor of hypoxia-inducible factor prolyl hydroxylases, has been found to stabilize HIF-1α expression under normoxic conditions in vitro and in vivo [13]–[15]. The dose of EDHB is usually 100 mg/kg body weight in mice and the main administration route is intraperitoneal injection [13], [20]. However, a dose of 250 mg/kg body weight EDHB has also been used in mice [15]. Therefore, because the dose of EDHB in rabbits is approximately 40–100 mg/kg body weight according to the dose conversion formula of mice to rabbit derived by the body surface area, we selected 50 mg/kg body weight as the EDHB dose in this study considering its potential side effects. H. Kasiganesan's study found that the systemic treatment of mice with EDHB, leads to elevated levels of HIF-1α in the liver and HIF-inducible EPO in serum [15]. In our study, HIF-1α and HIF-inducible VEGF expression measured by immunohistochemistry obviously increased two days after the first intraperitoneal injection of EDHB, indicating that EDHB therapy inhibited degradation of HIF-1α and promoted expression of VEGF in the femoral head.

Corticosteroids induce osteonecrosis of the femoral head through multi-system pathways [1]. Previous research has shown that corticosteroid disrupts the process of angiogenesis and the penetration of new vessels into necrotic bone [21], [22]. HIFs are crucial mediators of the adaptive cell response to hypoxia. It was confirmed that activating the HIF pathway promotes the expression of VEGF [23], [24] which was consistent with our results. We found that activating the HIF pathway by EDHB increased the expression of VEGF which is an important downstream target of HIFs. In addition to VEGF, HIF-1α could also induce the expression of angiogenic factors such as angiopoietin-1, angiopoietin-2, placental growth factor, and platelet-derived growth factor-B [25]. Considering that angiogenesis is a complex process and it is believed that the use of a single factor is insufficient to form functional vascular structures [26], activating the HIF pathway by EDHB which sequentially induces those angiogenic factors would be a promising way to promote new vascular formation. In Christina Warnecke's study showed that EDHB induces angiogenesis by HIF activation in a rat sponge model [27]. In our study, we analyzed microvessels by ink artery infusion angiography and microvessel density analysis after the immunohistochemical staining of CD31. This showed more blood vessels in the femoral heads of the prevention group and fewer blood vessels in the model group, indicating that EDHB neutralized the inhibitory action of corticosteroid on angiogenesis and promoted angiogenesis.

Corticosteroid also affects apoptosis. Studies have indicated that apoptosis partially causes bone cell death in osteonecrosis of the femoral head in patients [28]. Weinstein et al. showed that corticosteroid promotes apoptosis of osteoblasts and osteocytes and inhibits osteoblastogenesis in mice with glucocorticoid-induced osteoporosis [29]. In a rabbit model of steroid-associated necrosis of the femoral head, our TUNEL assay results showed that apoptosis indeed existed in osteocytes and bone marrow cells in the early stage of glucocorticoid-induced femoral head osteonecrosis. In the study of Tian L et al., they observed the similar results [30]. Bcl-2 is an anti-apoptotic protein, but caspase-3 is a pro-apoptotic protein. Both are important apoptosis-related proteins and play central role in the regulation of apoptosis. The expression level of caspase-3 increased significantly and the level of Bcl-2 decreased during the development of steroid-associated avascular necrosis of the femoral head [30], a finding that was also confirmed by our study. Previous studies found that HIF-1 regulates several apoptosis-related factors including both pro-apoptotic (e.g., BNIP3, NIX and NOXA) and anti-apoptotic factors (e.g., Bak, Bax, Bcl-xL, Bcl-2, Bid, Mcl-1, NF-κB, p53 and survivin) [31]–[34]. Chen MH et al. found that HIF-1α over-expression increased Bcl-2 expression and significantly reduced apoptotic cells in a rat spinal cord injury model [35]. In this study, the TUNEL assay and immunohistochemical staining showed that the apoptosis was decreased accompanied by increased Bcl-2 and decreased caspase-3 expression in the prevention group with EDHB administration, indicating that EDHB could interfere with apoptosis and prevent the occurrence of steroid-associated osteonecrosis.

Taken together, our findings suggest that systemically administrating EDHB prevent steroid-associated ONFH in rabbits by promoting angiogenesis and inhibiting apoptosis.

One of the limitations of the present study is that we only explored the effects of EDHB on apoptosis in bone cells and hematopoietic tissue and did not explored the underlying mechanism. The probable mechanisms are as follows: (1) EDHB promotes neovascularization and the newly formed blood vessels bring more nutrients and decrease apoptosis in bone cells and hematopoietic tissue; (2) EDHB regulates apoptosis-related factors through the HIF pathway; (3) EDHB promotes cellular metabolic flexibility by activating effects on the HIF pathway in cellular energy metabolism, which enhances apoptosis resistance [36]. In the near future, our group is planning to undertake additional studies to better clarify the mechanism of the effects of EDHB on apoptosis.

In conclusion, our results suggest that the administration of EDHB has the potential to become a novel and simple method to prevent the development of steroid-associated osteonecrosis.

## Supporting Information

Checklist S1 (PDF)
